# A Novel Approach for Comparing Selected Metabolites in Citrus Leaves and Fruits Across Datasets

**DOI:** 10.3390/plants14101406

**Published:** 2025-05-08

**Authors:** Ryan C. Traband, Xuesong Wang, Mariano Resendiz, Megan Meng, Yoko Hiraoka, Qiong Jia, Rendell Chang, Ethan Eurmsirilerd, Tracy Kahn, Peggy A. Mauk, Amancio De Souza, Anil Bhatia, Haiyan Ke, Donald Merhaut, Mikeal L. Roose, Zhenyu Jia, John M. Chater

**Affiliations:** 1 Department of Botany and Plant Sciences, University of California, Riverside, CA 92521, USA; ryan.traband@email.ucr.edu (R.C.T.); peggym@ucr.edu (P.A.M.); donald.merhaut@ucr.edu (D.M.);; 2 Genetics, Genomics, and Bioinformatics Program, University of California, Riverside, CA 92521, USA; 3 Metabolomics Core Facility, University of California, Riverside, CA 92521, USAhaiyanke@ucr.edu (H.K.); 4 Department of Horticultural Sciences, University of Florida, Lake Alfred, FL 33850, USA

**Keywords:** metabolomics, method, citrus, comparison, datasets

## Abstract

Citrus fruits are valued not only for their nutritional benefits but also for their rich phytochemical content. Metabolomics has emerged as a comprehensive technique for assessing the chemical composition of fruits. The botanical connection between leaves, flowers, and fruits is reflected in both their structure and chemical composition, particularly in the flow of nutrients between plant organs. We introduced a new logarithm ratio-based approach to compare metabolite profiles between fruits and leaves. We hypothesize that this method allows for the analysis of multiple citrus metabolomic profiles to reveal known and novel correlation patterns, reflecting the dynamic connections between metabolic sources. To test this hypothesis, we leveraged comprehensive leaf metabolomic profiles from over 200 accessions in the Givaudan Citrus Variety Collection and reviewed published metabolomics data for fruits and juices of matching citrus types. By employing logarithm-transformed metabolic ratios within each dataset, we accounted for systematic differences across metabolomic platforms, achieving an unbiased analysis.

## 1. Introduction

Citrus production holds significant importance in the agricultural sector on a global scale, with annual production at 158.5 million tons. As of 2021, the top citrus-producing countries include China, Brazil, India, and Mexico [1]. In the United States, citrus fruits account for 14% of the total fresh fruit volume, with oranges ranking fourth in terms of per capita consumption, behind bananas, apples, and melons. Highly valued for their flavor, vitamin C, lycopene, and high phenolic content, citrus fruits are also used for their distinct scents and oils, derived from the rind, in a variety of applications [2]. In light of these remarkable attributes, citrus, along with other fruits, serves as a veritable treasure trove of diverse phytochemicals, all synthesized within a single organism. In fact, plants outshine all other kingdoms on Earth by possessing the highest number and the most diverse set of secondary metabolites distributed across different parts of their bodies [3]. In the fruit industry, various metrics are employed to assess the chemical composition of fruits, including total soluble solids, total acidity, flavor, texture, and water content, using a variety of assays. However, metabolomics is a powerful tool for comprehensively analyzing plant biochemical diversity when used in sync with genetics or transcriptomics. Metabolomics is the study of all small molecules, known as metabolites, detected within a biological system. It provides insights into biochemical processes by analyzing the composition, concentration, and interactions of metabolites, reflecting the organism’s physiological state. This powerful but costly method uses techniques like mass spectrometry (MS) and nuclear magnetic resonance (NMR). Metabolites are either detected through targeted analysis where specific metabolites and their isomers can be quantified in terms such as mg/L and μg/g or untargeted analysis often measured in arbitrary units (a.u). Metabolomics helps uncover metabolic pathways, identify biomarkers, and understand how genetics and environmental factors influence metabolism.

Metabolomics offers valuable insights into metabolic pathways that contribute to species differentiation and bioactive compound accumulation. In citrus, metabolomics has been instrumental in identifying key genetic factors influencing flavonoid and coumarin biosynthesis. Recent large-scale studies, such as that by Xiao Liang (2024) [4], have mapped metabolic variation across diverse citrus accessions, linking thousands of genetic markers to metabolite accumulation patterns. Their findings highlight how distinct citrus species have undergone metabolic specialization, particularly in pathways governing flavonoid glycosylation and coumarin accumulation, which influence both the nutritional properties and potential pharmacological effects of citrus fruits. By integrating metabolomic and genomic data, researchers can better understand how selective breeding and evolutionary pressures have shaped citrus metabolite profiles, providing a foundation for developing varieties optimized for health benefits and reduced adverse effects [4]. This approach, using genetics and transcriptomics in conjunction with metabolomics, has been widely adopted in plant research. For instance, Wang et al. analyzed metabolomic profiles in various tissues of a single plant [5]. Yazici et al. compared the metabolic profiles of identical tissues among different genotypes [6]. Additionally, Asai et al. explored the variability in metabolite abundance within a specific tissue type across different species of citrus subjected to tissue wounding [7]. In this paper, we introduce a new log-ratio-based approach to compare metabolite profiles between fruits and leaves and across major citrus groups.

## 2. Materials and Methods

### 2.1. Fruit Datasets and Selection Criteria Used

Fruit datasets were chosen in terms of their ability to provide raw metabolomic data for Citrus fruit flesh, specifically juice or pulp. Data needed to be displayed in units such as (a.u.) for untargeted work and ng/mL, µg/mL, mg/L, µg/g, nmol/g, or a.u. for targeted work. Publications providing data exclusively in the form of principal components or in formats inaccessible unless through paid or outdated software were also excluded. Shi Feng et al. (2018) focused on the metabolite profiles responsible for how preharvest factors, including genotype, rootstock, and grove location, influence the quality of mandarin fruit [8]. Volatile analysis was carried out on a Clarus 680 gas chromatograph (GC) (PerkinElmer, Inc., Waltham, MA, USA) equipped with a Clarus SQ 8T mass spectrometry (MS) detector. The publication provided a broader list of metabolites but focused exclusively on only four varieties of mandarin hybrids [8]. The four varieties studied were Clementines (*Citrus clementina* hort. ex Tanaka), Page (*Citrus tangelo*), Daisy (*Citrus reticulata* Blanco), and Satsumas (*Citrus unshiu* Marcovitch). Each variety had fruit collected from three different locations: Clementines were sourced from Los Angeles, CA, USA, Pasadena, CA, USA, and Fowler, CA, USA, Page mandarins were sourced from Fowler, CA, USA, and Daisy Mandarins were from Lindcove, CA, USA. For each variety, three fruits were sampled to prepare the juice, flesh, and other tissues. Further details on replicates were not specified.

Wang S (2016) analyzed 47 different cultivars of citrus using a non-targeted metabolomics approach [5]. They detected more than 2000 metabolite signals, from which more than 54 metabolites, including amino acids, flavonoids, and limonoids, were identified/annotated to understand the spatial and temporal distribution of metabolites in different species and different tissues. The 47 accessions were collected from the following species: lemon (*Citrus lemon* [L.] Burm f.), pummelo (*Citrus grandis* (L.) Osbeck), grapefruit (*Citrus paradisi* Macf), sweet orange (*C. sinensis* [L.] Osbeck), and mandarin (*C. reticulata* Marcf.). Cultivars were harvested randomly from trees in three positions and pooled for each biological replicate. The fruits were washed and the tissues of the flavedo, albedo, segment membrane (SM), and juice sacs (JS) were separated.

In a paper published by Shouchuang Wang in 2024, a comprehensive metabolic profiling of various citrus species from different locations was performed, and metabolic profiles were compared among the species, with a focus on the phenylpropanoid metabolic pathway [9]. In this study, a total of 189 citrus germplasm resources of different types were collected, mainly including 154 pummelos, as well as 15 mandarins, 15 oranges, 1 lemon, 1 citron, and 1 papeda. For the reasonableness of sampling, five to ten mature fruits around the crown of a tree were picked from three random positions per tree as biological replicates. Two biological replicates per cultivar were collected. The structures of 360 metabolites were identified by comparing the mass spectral fragments and retention times of standards, by the manual annotation of mass spectral profiles, and by searching metabolic databases; these included primary metabolites of amino acids and vitamins, as well as secondary metabolites such as lipids and phenylpropanoids. A multiple reaction monitoring method based on a triple quadrupole–linear ion trap mass spectrometer (API 4000, AB Sciex, Framingham, MA, USA) was used for the quantitative analysis of metabolites and for accurate time-of-flight mass spectrometry. More specific information on these datasets can be found in their respective publications.

Wang (2017) [10] analyzed 62 Citrus accessions from a collection of popular/local cultivars in China. Twelve to twenty-one healthy fruits true to their cultivars and at commercial maturity were randomly collected from the peripheral canopies of at least three trees and were randomly divided into three biological replicates [10]. The washed material was separated into four tissues, including flavedo, albedo, SM, and JSs, and immediately placed in liquid nitrogen. Qualitative metabolic analysis via HPLC-DAD-ESI-QqTOF-MS/MS (6520B, Agilent, Santa Clara, CA, USA) was performed in the targeted MS2 mode. The UV spectra (DAD) were recorded from 270 to 380 nm. The raw data were analyzed using MassHunter software (https://www.agilent.com/en/promotions/masshunter-mass-spec) and the processing method was the same as previously described 32. Quantitative analysis of metabolites was carried out in the multiple reaction monitoring (MRM) mode by LC-ESI-Q TRAP-MS/MS (4000Q TRAP, ABI, Los Angeles, CA, USA). For a list of samples used from all datasets, please refer to Supplementary Table S1.

### 2.2. Leaf Dataset

Leaf material was collected from the Givaudan Citrus Variety Collection on a single day in both 2020 and 2022, with sampling from different parts of the canopy in all cardinal directions. In 2020, due to limited research activity during the COVID-19 lockdown, leaves were only collected from 60 varieties, with 2 trees sampled per variety, totaling 120 trees. This included the control varieties Clementina de Nules and Parent Washington, sampled at 9 am, 10 am, and 11 am. In 2022, approximately 230 varieties were sampled using the same strategy, including the same controls. Collected leaves were immediately frozen in liquid nitrogen and stored at −80 °C until analysis. Sample preparation involved freeze-drying and coarse grinding of the leaves with a mortar and pestle, followed by fine grinding using a bead beater. Approximately 10 mg of the finely ground powder was extracted using a monophasic solvent mixture. The extracts were then sonicated, vortexed, and centrifuged, and the supernatants were collected for further analysis.

For polar metabolite analysis, we used a TQ-XS triple quadrupole mass spectrometer coupled with an Acquity I-class UPLC system fitted with a ZIC-pHILIC column (made by Waters Corporation, Milford, MA, USA), following the protocols of Vliet et al. (2019) [11]. The system operated in MRM mode with optimized source and desolvation temperatures, gas flows, and capillary voltage. Quality control samples, prepared by pooling aliquots from all study samples, were used to ensure system stability.

Secondary metabolite analysis was performed using a Synapt G2-Si quadrupole time-of-flight mass spectrometer coupled with an Acquity I-class UPLC system, equipped with a CSH phenyl–hexyl column, as described in established protocols [10]. The mass spectrometer operated in a data-dependent acquisition mode, scanning a mass range of 50 to 1200 *m/z*. Quality control was ensured through the periodic analysis of pooled samples and the use of Leucine enkephalin for mass correction. All samples were analyzed in a randomized order to maintain consistency in data acquisition.

### 2.3. Log-Ratios of Two Metabolites Under Analysis

One advantage of this method is the ability to draw insights from multiple studies in which the abundance of metabolites was profiled for citrus fruits and leaves. However, a significant challenge lies in the potential systematic differences between samples or studies, including variations in assay platforms and other uncontrollable variables. To tackle this issue, we calculated the logarithm base 2 ration, *R*_AB_, of two selected metabolites within each sample, as specified in Equation (1) below:

(1) $$R_{AB}={log}_{2}\left(\frac{M_{A}}{M_{B}}\right)={log}_{2}\left(M_{A}\right)-{log}_{2}\left(M_{B}\right),$$

where *M_A_* and *M_B_* are the absolute abundances of metabolites *A* and *B*, respectively, and *R_AB_* is the *log*_2_ ratio of these two abundance values. Logarithm transformation is necessary due to the non-Gaussian distribution of the absolute abundance values for the metabolites, and using the ratio of two metabolite abundances is a special form of normalization that eliminates sample-specific or study-specific biases. Indeed, ratios of molecules have been widely utilized as metric scales in fruit research. For example, the ratio of total phenolics to antioxidant capacity is essential for determining what portion of the phenolics is responsible for the antioxidant activities of fruits [12]. Additionally, the ratio of malic acid to citric acid is commonly used to numerically determine a fruit’s flavor profile, ripeness, its value in fermentation processes, and more [13]. Through this bias correction step using log-ratio, the *R_AB_* value of these two selected metabolites can be calculated across samples and datasets for subsequent analysis and comparison. This approach is being used for the first time to investigate profiles in distinct plant organs across various accession groups (“accession groups” in this case meaning heavily related varieties and species that do not bear the same name or direct parentage), leveraging different data sources.

### 2.4. Statistical Methods

#### 2.4.1. Standard Quantitative Methods

Bar plots were used to illustrate the comparison of single metabolite ratios between leaf and fruit datasets, determined using Equation (1). Two-sided *t*-tests were used to compare the log ratios of metabolite pairs. To visualize metabolite profile comparisons across accession groups, datasets, and organs (leaf vs. fruit) using the log-ratio approach, we developed two volcano plot-based visualizations, as described below.

#### 2.4.2. Single Log-Ratio Analysis

This display only investigates the log-ratio of two selected metabolites *A* and *B* (a potential log-ratio signature) and surveys the mean log-ratio, i.e., $\bar{{log}_{2}\left(\frac{M_{A}}{M_{B}}\right)}$, of replicated samples for each accession group in each dataset. Each data point represents the statistics calculated from the replicates of an accession group in a dataset (representing leaf or fruit samples). For each data point, a one-sample *t*-test was performed to determine whether the mean ${log}_{2}\left(M_{A}\right)$ is significantly different from the mean ${log}_{2}\left(M_{B}\right)$. The x-axis shows the mean of the log-ratios (or the difference between the mean ${log}_{2}\left(M_{A}\right)$ and the mean ${log}_{2}\left(M_{B}\right)$), while the y-axis indicates the -*log*_10_ (*p*-value), where the *p*-value is calculated from the one-sample *t*-test. This analysis can be used to check (1) the repeatability of log-ratios in the same tissue type of the same accession groups across datasets, (2) the variation in or correlation of log-ratios between different tissues (leaf versus fruit) of the same accession groups across datasets, and (3) the general pattern of log-ratios between tissue types across accession groups and datasets.

#### 2.4.3. Analysis of Multiple Log-Ratios Between Leaves and Fruits

This display compares the log-ratios of multiple selected metabolite pairs (potential log-ratio signatures) and the means of the corresponding mean log-ratios between organs (leaf versus fruit in this study) for each accession group. Note that there are multiple fruit datasets but only one leaf dataset (our data) in this study. We treated the fruit datasets as replicates and determined whether the mean of the mean log ratios for these replicates is significantly different from the mean log ratio of the single leaf dataset using a one-sample *t*-test. Each data point represents the statistics for a metabolite pair (a potential log-ratio signature) calculated from the replicates of fruit datasets for an accession group, compared to the leaf dataset. The x-axis shows the difference between the mean of the mean log-ratio of the metabolite pair in fruit samples across datasets and the mean log-ratio in the leaf samples in our dataset, as shown in Equation (2):

(2) $$\frac{1}{n}\sum_{i=1}^{n}\bar{{log}_{10}\left(\frac{M_{Ai}}{M_{Bi}}\right)}-\bar{{log}_{10}\left(\frac{{M\prime }_{A}}{{M\prime }_{B}}\right)}$$

where *n* is the number of replicates of fruit datasets, *M_Ai_* and *M_Bi_* are the abundances of metabolites *A* and *B* in the *i*th replicate fruit dataset (with *i* = 1…*n*), and *M*’*_A_* and *M*’*_B_* are the abundances of metabolites *A* and *B* in the leaf dataset. The y-axis indicates the -*log*_10_ (*p*-value), where the *p*-value is calculated from the one-sample *t*-test. This analysis can be used to identify metabolite ratio signatures that are either similarly or differentially represented between plant organs, such as fruit versus leaf, in specific citrus accession groups or across accession groups.

## 3. Results and Discussion

### 3.1. Expected Similar Metabolites

**Figure 1:**
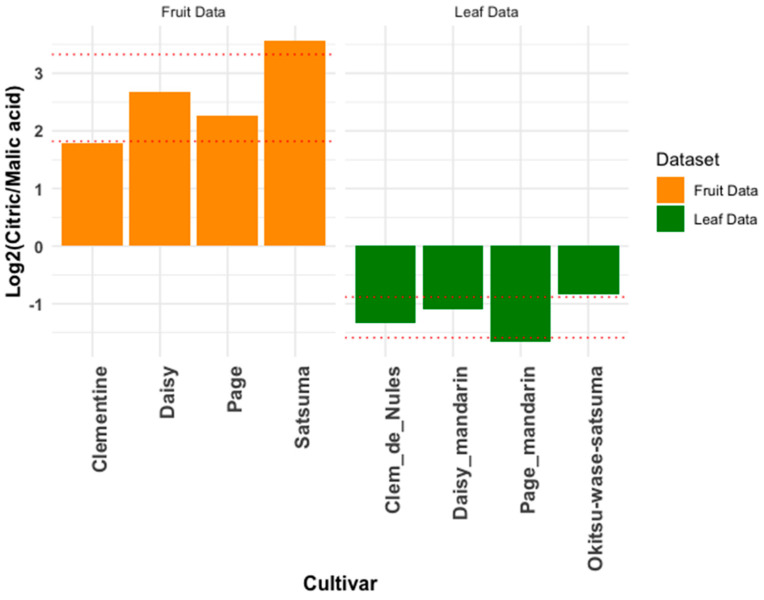
Citric-acid-to-malic-acid ratio in citrus fruit and leaves. This figure compares the citric-acid-to-malic-acid-ratio in the fruits and leaves of four mandarin varieties. Fruit data were sourced from Feng et al. (2018) [8], while leaf data were collected from the Givaudan Citrus Variety Collection at UC Riverside, representing similar but not identical mandarin varieties. Because each sample lacked replicates, error bars could not be calculated. The red dotted lines represent the standard error of the existing samples displayed on the chart.

Feng [8] analyzed the fruit metabolomes of four mandarin varieties using a targeted panel. In our study, we extracted metabolite profiles from the leaves of four similar varieties for comparison. Figure 1 illustrates the ratios of citric and malic acids, showing higher levels of citric acid in the fruit and higher levels of malic acid in the leaves. This difference may stem from the fruit’s need for more energy, as citric acid in the Krebs cycle generates guanosine triphosphate (GTP) and provides intermediates for amino acids and fatty acids. Citrus fruits also produce excess citric acid in the vacuoles of their juice sac cells. The vacuoles are relatively enlarged, and the tonoplast allocates citrate into the tonoplast through vacuolar H-ATPase and H-pyrophosphatases. The movement and maintenance of pH in these vacuoles creates the rapid acidification we taste in citrus [14,15]. In leaves, the lower levels of citric acid could be due to two main factors. First, citric and malic acids are more balanced in leaves, with malic acid playing a critical role in the Calvin cycle during photosynthesis, including the CAM, C4, and C3 pathways. Second, malic acid is essential for the water stress responses [16].

**Figure 2:**
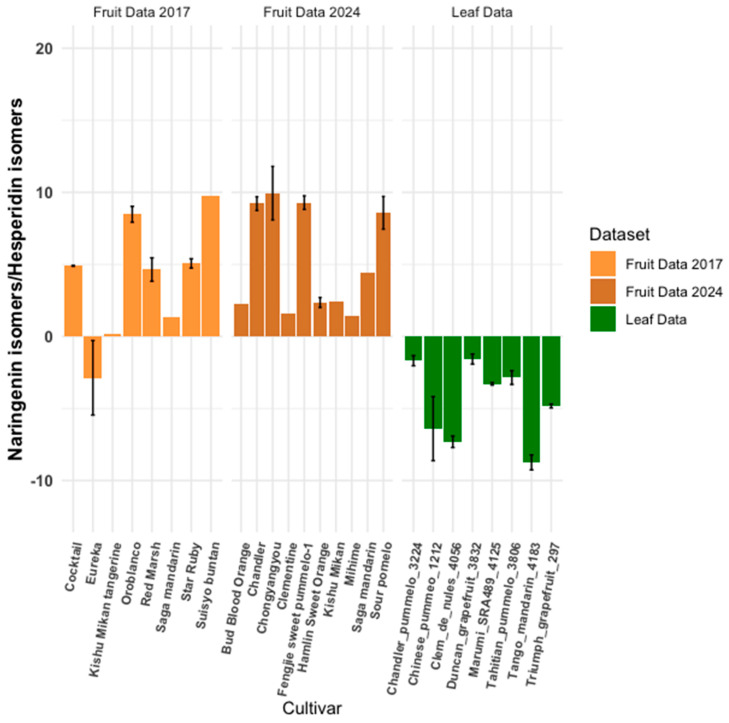
Naringenin-to-hesperidin ratio comparison. This figure compares naringenin-to-hesperidin ratios between a leaf metabolomics dataset and two fruit datasets, calculated using Equation (1) (Section 2). Error bars are unique to each sample. Samples without error bars lacked sufficient replicates.

Naringenin, a bitter molecule present in citrus and various plants, shares a biosynthetic pathway with hesperidin, another flavonoid. Despite their similarity, naringenin is notably more concentrated in pummelo and grapefruit than other citrus varieties and lines. Hesperidin is abundant in citrus fruits, particularly those referred to as “hesperidium fruits.” Their flavonoid profile, including the naringenin-to-hesperidin ratio, serves as a valuable chemotaxonomic marker for classifying and distinguishing citrus species and cultivars. Understanding the flavonoid composition of these molecules is crucial for assessing their potential nutritional benefits to humans and for guiding breeding programs and cultivar identification. As anticipated, pummelo and grapefruit cultivars consistently exhibit significantly higher naringenin than hesperidin concentrations across all datasets (Figure 2). Interestingly, certain mandarin varieties have been observed to contain higher quantities of hesperidin compared to other cultivars [17,18].

**Figure 3:**
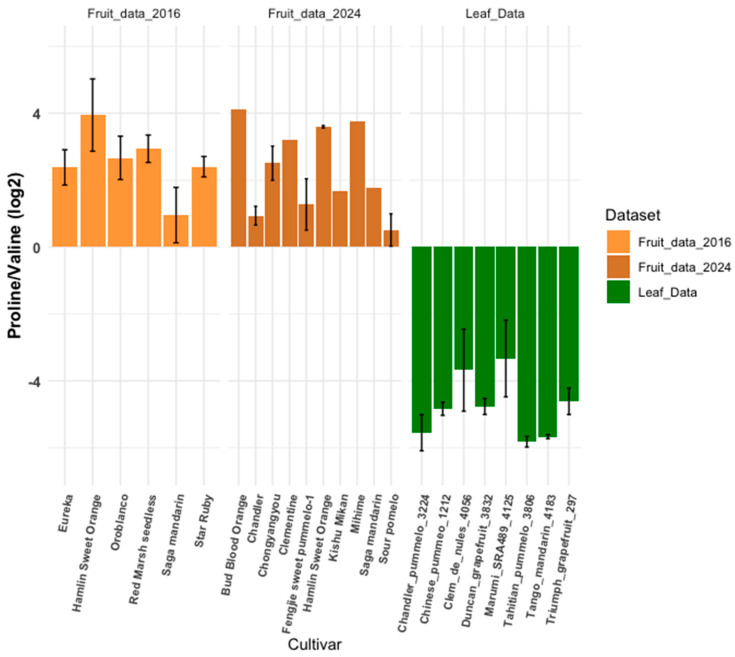
Proline-to-valine ratio comparison. This figure compares proline-to-valine ratios between a leaf metabolomics dataset and two fruit datasets, calculated using Equation (1) (Section 2). Error bars are unique to each sample. Samples without error bars lacked sufficient replicates.

Unlike mammals, plants synthesize their own aromatic amino acids through the shikimate pathway. Analyzing amino acid ratios can provide insights into a plant’s metabolic state at the time of collection or reveal differences between treatment groups or related individuals. For instance, the proline-to-valine ratio serves as an indicator of abiotic stress. Proline accumulates in plants under drought, salinity, and extreme temperatures due to its roles as an osmoprotectant, protein and membrane stabilizer, and free radical scavenger. Figure 3 shows a distinct difference in the proline-to-valine ratio between leaves and fruit, suggesting a potential developmental response related to late fruit maturation. This observation is consistent with findings in Hayet et al. [17], which examined proline responses in grapevines. It is noteworthy that the proline in fruit likely results from maturation signals involving ornithine rather than stress-induced accumulation through glutamine [19].

### 3.2. Expected Similar Metabolite Ratios

**Figure 4:**
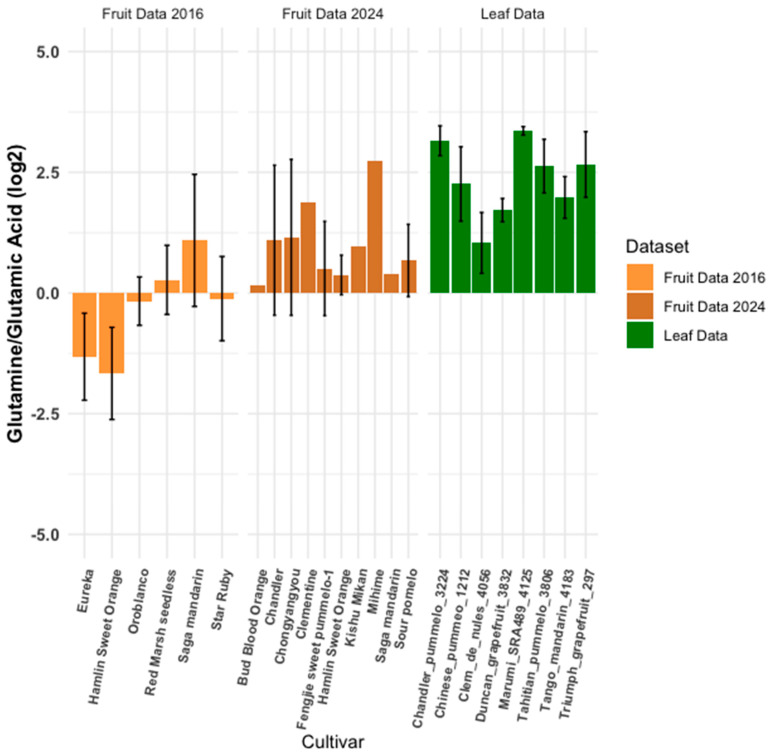
Glutamate-to-glutamic-acid ratio comparison. This figure compares glutamate-to-glutamic-acid ratios between a leaf metabolomics dataset and two fruit datasets, calculated using Equation (1) (Section 2). Error bars are unique to each sample. Samples without error bars lacked sufficient replicates.

Essential building blocks like the 20 amino acids and ubiquitous plant compounds such as quercetin [20] are consistently present across organisms. The stability of these common metabolites within the ratio model highlights the method’s robustness, even in the face of dataset variability. Glutamine and glutamic acid, closely related compounds differing by a single amide group, are key players in plant stress responses and amino acid synthesis. These amino acids are vital for protein synthesis, influencing plant growth and development, and their abundance and interconversion affect the overall amino acid pool. Figure 4 shows the glutamine-to-glutamate ratio across three databases from a subset of mandarins and pummelos. While some variance and outliers exist, a general trend of similar ratios appears across both fruit juice and leaf tissue datasets, reflecting relative stability. Ideally, log ratios should center around zero. This is in stark contrast to the naringenin-to-hesperidin ratios (Figure 2) and citric-to-malic-acid ratios (Figure 1), which exhibit more distinct behaviors. Overall, the variance in Figure 4 is relatively low, with glutamine-to-glutamate ratios ranging from approximately −2 to 2, likely due to environmental factors and genetic differences among citrus varieties. Given their structural similarity, these amino acids are expected to be consistently abundant across plant tissues. However, as demonstrated in Figure 2, stronger differences appear when different species and cultivar groups are compared.

### 3.3. Metabolite Ratios: Intra-Cultivar-Group Comparison

**Figure 5:**
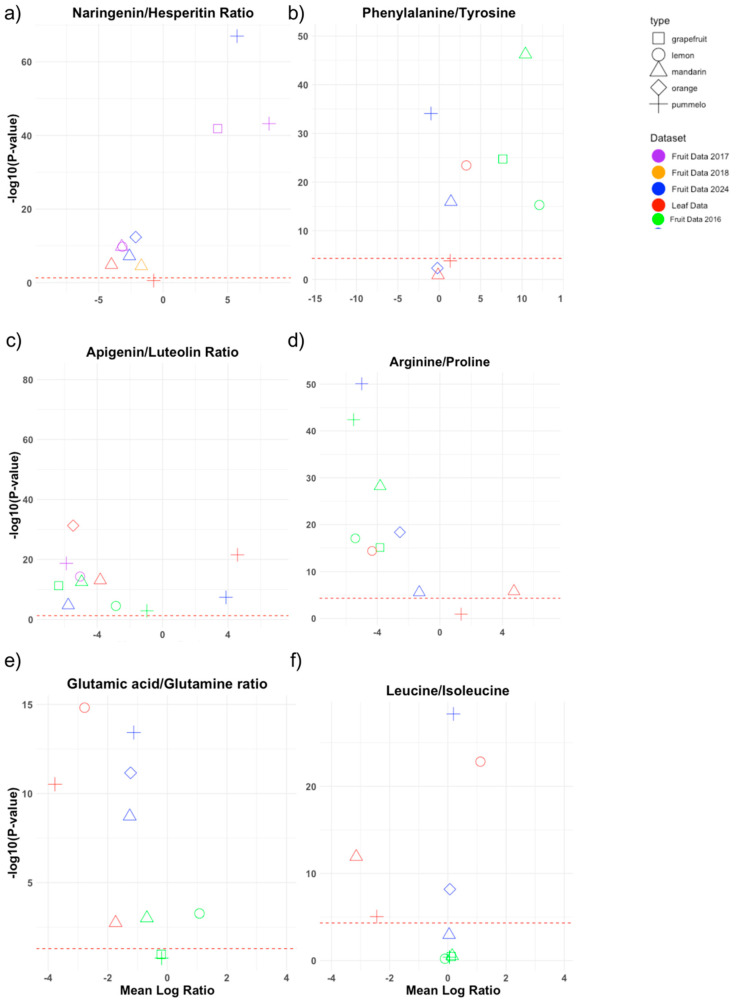
Volcano plot comparing metabolite ratios across multiple datasets (including fruit and leaf tissues) and citrus categories. The x-axis represents the mean log ratio of two selected metabolites, with negative values indicating a higher abundance of the denominator metabolite and positive values indicating a higher abundance of the numerator metabolite. The y-axis represents the significance level for the difference in the mean log ratio from 0, with a threshold set at -*log*_10_(0.05) (one-sample *t*-test). (**a**) Examines the ratio between naringenin hesperitin. (**b**) Examines the ratio between phenylalanine and tryosine. (**c**) Examines the ratio between apigenin and luteolin. (**d**) Examines the ratio between arginine and proline. (**e**) Examines the ratio between glutamic acid and glutamine. (**f**) Examines the ratio between leucine and isoleucine.

We adapted the volcano plot, typically used in differential gene expression analysis, to compare metabolite ratios between fruits and leaves across major citrus cultivar groups. Figure 5 illustrates six distinct metabolite ratios, derived from three fruit-based and one leaf-based dataset. This plot integrates the significance and association of different tissues, species, and datasets into a single chart. Two of the selected ratios, glutamate to glutamic acid and proline to valine, represent homeostatic relationships, reflecting the structural similarity of these metabolites. The figure highlights both expected similarities and unique characteristics of each metabolite ratio.

The naringenin-to-hesperidin ratio, as previously observed, is visualized with a broader range of varieties across samples (Figure 5a). Pummelo and grapefruit fruit samples consistently exhibit higher levels of naringenin compared to hesperidin, while pummelo leaf data demonstrate lower levels, distinct from other leaf and fruit data points, confirming a known trend. The glutamate-to-glutamine and phenylalanine-to-tyrosine ratios (Figure 5b) exhibit remarkable stability, with data points clustering according to their respective datasets, indicating minimal variation beyond data source differences. This is expected, as phenylalanine is a precursor to tyrosine, and glutaminase catalyzes the conversion of glutamine to glutamate. Although cultivar groups show no discernible trends, comparing leaf and fruit data reveals higher glutamine levels in leaves and higher glutamic acid levels in fruit datasets.

The apigenin-to-luteolin ratio (Figure 5c), a comparison of two flavonoids, can reflect a plant’s response to various stresses, including UV radiation, drought, or pathogen attack. Flavonoids serve as antioxidants and UV protectants, and their balance may indicate a plant’s resource allocation for different stress responses. In Figure 5a, mandarins generally exhibit a higher luteolin-to-apigenin ratio, suggesting greater activity of flavone synthase (FNS) and possibly other enzymes like flavonoid 3′-hydroxylase (F3′H), which catalyze the hydroxylation step necessary for luteolin production. Conversely, pummelos tend to produce more apigenin, indicating higher FNS activity but lower F3′H activity, resulting in apigenin, which lacks the additional hydroxyl group found in luteolin. Figure 5d indicates that the arginine-to-proline ratio (Figure 5d) generally favors proline in fruit but arginine in leaves. Leaves might prioritize arginine for protein synthesis and nitrogen assimilation, essential for growth and metabolism. Fruit’s preference for proline could indicate an adaptation to osmotic stress or a nitrogen storage mechanism readily convertible to arginine [21,22]. The glutamine-to-glutamic-acid ratio (Figure 5e) has all datasets clustering within their respective colors (dataset), meaning that there is a distinct difference between datasets but not between these different cultivar groups. Glutamine and glutamic acid often show relatively consistent levels across various conditions due to their crucial functions in nitrogen assimilation and transport [23,24]. Figure 5f, covering the leucine-to-isoleucine ratio, has a large clustering in one area, with some variability. Leucine is clearly favored because it tends to accumulate better than isoleucine [25,26], which follows what is found in most studies in the literature covering these amino acids in organisms. The ratio of leucine to isoleucine varies based on species, environmental conditions, and growth stages, with leucine often present in higher concentrations due to its role in growth regulation and biomass accumulation. This balance is crucial for protein synthesis, the stress response, and metabolic stability, as both amino acids contribute to energy metabolism and resilience against environmental stress.

### 3.4. Metabolite Ratios: Inter-Cultivar Group Comparison

**Figure 6:**
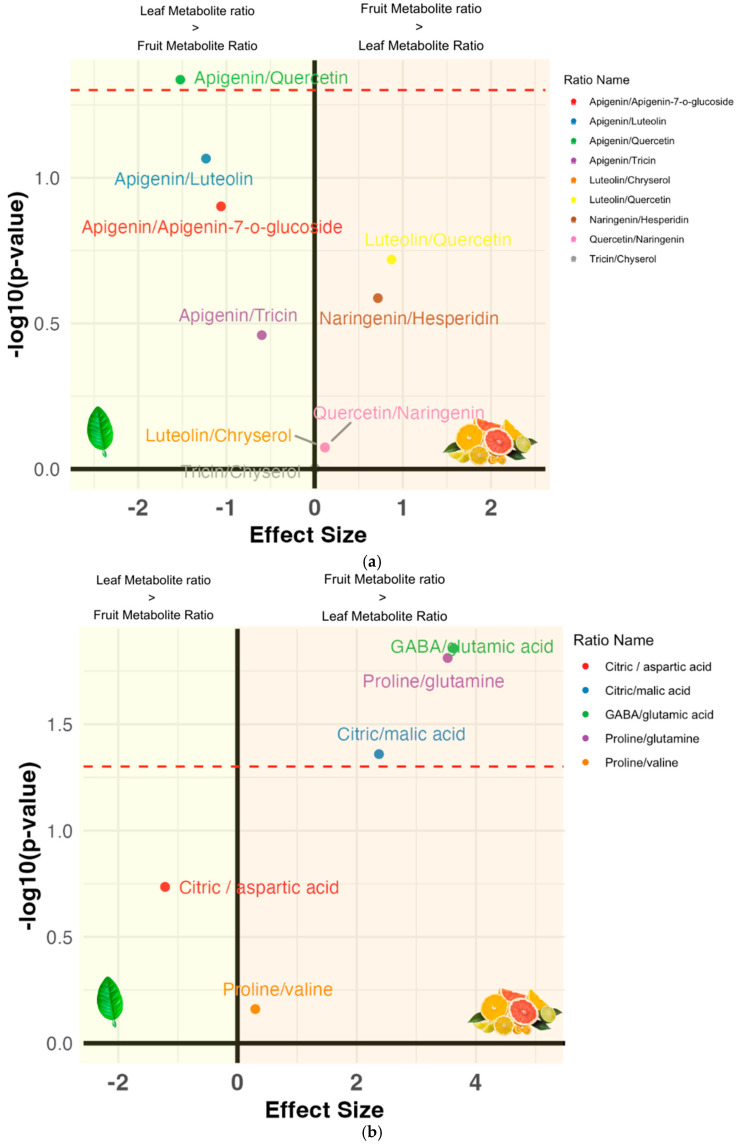
(**a**) Volcano plot of untargeted metabolites (flavonoids): This plot identifies significant differences in flavonoid ratios between fruit and leaf datasets. The x-axis represents the difference in average log ratios between fruits and leaves, with positive values indicating higher log ratios in fruits and negative values indicating higher log ratios in leaves. The y-axis represents the significance level, with a threshold of -log_10_(0.05). Each data point represents a specific flavonoid ratio. (**b**) Volcano plot of targeted metabolites (amino acids and common acids): This plot identifies significant differences in ratios of targeted metabolites (amino acids and common acids) between fruit and leaf datasets. The x-axis and y-axis interpretations are the same as in Figure 6a. See Equation (2) for the calculation [28].

We then compared the selected metabolite ratios of the combined fruit datasets to our leaf dataset. The volcano plots in Figure 6 indicate which metabolite is more abundant in which plant organ. For example, Figure 6a shows a significant preference for the citric-to-malic-acid ratio in fruit. The citric-to-aspartic-acid ratio, in this context, is used as a placeholder to compare citric acid to another common organic acid to confirm its abundance. More common protein ratios (Figure 6a), such as proline to alanine, exhibit lower significance and are more balanced, hovering close to zero on the x-axis. The proline vs. glutamate ratio indicated higher proline levels in citrus fruit, which often result from environmental stress. Proline acts as an osmoprotectant, helping cells manage drought, salt, and temperature extremes. Proline stabilizes proteins and provides energy for recovery, while glutamine focuses on nitrogen transport, making it less responsive to immediate stress. In Figure 6b, several phenolic ratios are compared to apigenin as a reference, with each ratio ascending to a higher level of significance. Only apigenin and quercetin exceeded the significance threshold in Figure 6b. Apigenin and quercetin are flavonoids, which are often associated with plant stress responses and defense. The higher, significant apigenin/quercetin ratio in leaves may reflect the leaves’ role in synthesizing or storing such compounds to protect the plant [27]. While Figure 6b lacks significant findings in this series, it is intentionally designed to demonstrate how to analyze the relationship between a specific metabolite (like apigenin) and a list of other metabolites. With specific targets and congruent datasets, entire pathways can be plotted in this same manner.

Genomics and transcriptomic data are often favored over metabolomics data, largely due to the larger sample sizes and greater potential for extensive biological analysis and collaboration. Metabolomics, however, is inherently dynamic—its variability across tissues, organisms, and environments, along with differences in analytical platforms, presents challenges for large-scale data collection and analysis. Consequently, many metabolomics studies are limited by a small number of replicates and a narrow focus on specific metabolites, and it is challenging to combine data from different studies for joint analysis [29]. Despite these obstacles, we have developed an approach to assess citrus metabolomics databases, integrating numerous samples across different organs, diverse varieties, and multiple studies, representing a significant advancement in the field. We propose a logarithmic ratio of selected metabolites to mitigate systematic differences across studies, enabling the joint analysis of data focused on different crop parts to test relevant hypotheses. Although the datasets (from different studies) included in this analysis were originally designed for specific objectives, they offer valuable data for broader comparative analyses. The strength of this approach lies in identifying large-scale trends across multiple datasets, allowing informed decision-making before embarking on costly new metabolomic experiments. While comparing logarithmic ratios is effective, it has limitations—ratios can amplify trends, and log transformations may distort the biological relevance of small differences. Nonetheless, this method offers a normalized and statistically manageable framework to analysis of metabolites.

Comparing metabolite profiles between different plant organs, such as citrus fruits and leaves, provides insights into molecular transport dynamics and the reasons for specific molecular enrichment in certain organs [30,31]. Genetic factors likely drive the observed variation in metabolite enrichment across cultivars. Identifying these genetic determinants will clarify the functions of associated genes and pathways and their roles in different plant tissues. Chemotaxonomic markers and their genetic counterparts can be developed to help identify promising lines for breeding and transformation [32].

Large databases, like the one created in the publication by Xiao Liang (2024) [4], are an excellent example of comprehensive reference databases that can be established to support future metabolomic comparisons, potentially transforming citrus breeding by enabling the prediction of fruit traits based on metabolite or genetic profiles from young leaf samples. In the publication, through a metabolite-based genome-wide association study (mGWAS) and strong validation, they discovered two loci key to UDP-glycosyltransferase (UGT) and glycosylation. This kind of discovery allows breeders to select for desirable traits and optimal parental lines, expediting cultivar development. However, methods like log ratio transformation could be the first step in connecting these databases to others for the discovery of even more loci. In this study, we demonstrated an approach using a few selected metabolite ratios; future research could expand this by using Procrustes analysis to explore other important ratios between fruits, leaves, and additional organs, such as flowers, given the available data. For example, comparing citric-to-malic-acid or glucose-to-fructose ratios could offer insights into sugar and acid levels [33,34], while tannin or saponin ratios might serve as indicators of bitterness. Establishing standardized practices for data collection, storage, and metabolite profiling across institutions is also essential to foster collaboration, data sharing, and robust analysis in the field.

## 4. Conclusions

We introduced a novel log-ratio-based approach to compare metabolite profiles between fruits and leaves across major citrus groups. This method revealed both distinguishing relationships—such as those involving citric and malic acids—and minimal differences, as seen with glutamic acid and glutamine. We used known metabolite relationships, like naringenin and hesperidin, to show the approach can come to similar conclusions as the scientific literature. Further, we demonstrated that the method is able to compare multiple ratios at once across just two datasets. This method has the advantage of using metabolite log-ratios for the reduction in inter-study bias, allowing smaller datasets from various sources to be combined for analyses and thereby enhancing the statistical power to test diverse hypotheses. This method holds promise for standardizing metabolite profiles in different studies with similar research directions across other crops or organisms, including humans.

## Data Availability

The original contributions presented in this study are included in the article/Supplementary Materials. Further inquiries can be directed to the corresponding author(s).

## Supplementary Materials

The following supporting information can be downloaded at: https://www.mdpi.com/article/10.3390/plants14101406/s1.
